# A novel model associated with tumor microenvironment on predicting prognosis and immunotherapy in triple negative breast cancer

**DOI:** 10.1007/s10238-023-01090-5

**Published:** 2023-05-23

**Authors:** Juan Zhang, Mi Zhang, Qi Tian, Jin Yang

**Affiliations:** 1 Cancer Center, The First Affiliated Hospital of Xi’an Jiaotong University, Xi’an, China; 2 Precision Medicine Center, The First Affiliated Hospital of Xi’an Jiaotong University, Xi’an, China; 3 Department of Medical Oncology, The First Affiliated Hospital of Xi’an Jiaotong University, 277 Yanta Western Road, Xi’an, 710061 Shaanxi China

**Keywords:** Triple-negative breast cancer (TNBC), TCGA, Tumor immune microenvironment (TIME), Prognosis, Immunotherapy

## Abstract

Triple negative breast cancer (TNBC) is the most aggressive and malignant subtype in breast cancer. Immunotherapy is a currently promising and effective treatment for TNBC, while not all patients are responsive. Therefore, it is necessary to explore novel biomarkers to screen sensitive populations for immunotherapy. All mRNA expression profiles of TNBC from The Cancer Genome Atlas (TCGA) database were clustered into two subgroups by analyzing tumor immune microenvironment (TIME) with single sample gene set enrichment analysis (ssGSEA). A risk score model was constructed based on differently expressed genes (DEGs) identified from two subgroups using Cox and Least Absolute Shrinkage and Selector Operation (LASSO) regression model. And it was validated by Kaplan–Meier analysis and Receiver Operating Characteristic (ROC) analysis in Gene Expression Omnibus (GEO) and the Molecular Taxonomy of Breast Cancer International Consortium (METABRIC) databases. Multiplex immunofluorescence (mIF) and Immunohistochemical (IHC) staining were performed on clinical TNBC tissue samples. The relationship between risk score and immune checkpoint blockades (ICB) related signatures was further investigated, as well as the biological processes were performed by gene set enrichment analysis (GSEA). We obtained three DEGs positively related to prognosis and infiltrating immune cells in TNBC. Our risk score model could be an independent prognostic factor and the low risk group exhibited a prolonged overall survival (OS). Patients in low risk group were more likely to present a higher immune infiltration and stronger response to immunotherapy. GSEA revealed the model was associated with immune-related pathways. We constructed and validated a novel model based on three prognostic genes related to TIME in TNBC. The model contributed a robust signature that could predict the prognosis in TNBC, especially for the efficacy of immunotherapy.

**Supplementary Information:**

The online version contains supplementary material available at 10.1007/s10238-023-01090-5.

## Background

Breast cancer is a tumor with a high prevalence rate in women [1], and its incidence and mortality are showing an upward trend year by year. The latest global cancer burden data in 2020 shows that breast cancer surpasses lung cancer to become the largest cancer in the world [2]. TNBC is one of the subtypes of breast cancer, which occupies about 15–20%. The prognosis of TNBC is extremely worst with the character of highest histological grade and rates of metastasis [3, 4]. Due to the lack of expression of estrogen receptor, progesterone receptor and human epidermal growth factor receptor 2 (HER-2), TNBC is not sensitive to endocrine therapy and HER-2 targeted therapy.

With the emergence of large-scale sequencing and other multi-omics technologies such as gene expression profiling, proteomics, immune omics and other detection methods, it has been proved that TNBC has stronger heterogeneity [5]. TNBC is no longer a kind of disease, but a collection of diseases [6–10]. In response to the heterogeneous feature of TNBC and to achieve precise treatment of tumors, different subtypes should be given different treatments according to the biological characteristics of each subtype. At present, immunotherapy is a hot spot in cancer which treats tumors by regulating the immune system response. Studies showed that immunotherapy may have a better effect on highly immunogenic tumors (inflammatory tumors or "hot" tumors) [11]. TNBC obtains unique biomolecular characteristics and higher immunogenicity than other types of breast cancer [12, 13]. A large number of clinical trials confirmed that TNBC patients who received immunotherapy and responded well could have a better prognosis and long-lasting benefits. The classic results of KEYNOTE-522 study [14] and Impassion130 study [15] suggested that TNBC could benefit from immunotherapy in both early and advanced stages. Immunotherapy is emerging as a promising and effective treatment option for TNBC. However, not all TNBC patients were sensitive to immunotherapy. In order to maximize the benefits of treatment and reduce the side effects of drugs, it is necessary to screen out the patients with a sensitive response to immunotherapy [16].

The tumor microenvironment (TME) plays an important role in tumorigenesis and tumor progression of many cancers, which is related to the prognosis of patients [17]. Cancers develop through continuous interaction between tumor cells and various immune cells in TME. Stimulating a sustained antitumor immune response is the key factor to achieve the clinical benefits of immunotherapy [18]. Tumor-infiltrating lymphocytes (TILs) as an important part of TME were considered closely related to efficacy of immunotherapy and favorable prognosis in breast cancer [19, 20]. But the accuracy and manifestation of TILs were found to be limited [21].

In this study, we systemically portrayed the TIME in TNBC by analyzing the mRNA sequencing profiles from TCGA database. After clustering all samples into two subgroups, we distinguished three DEGs related to immune infiltration and prognosis of patients. A risk score model was constructed based on the DEGs to predict the survival of patients and the efficiency of immunotherapy in TNBC. We further validated the risk score model by GEO and MATABRIC cohorts. Furthermore, we determined the risk score to be an independent prognostic signature that could accurately predict the 3- and 5-year OS of patients with TNBC by ROC analysis. We also investigated the correlation between the model and proportions of infiltrating immune cells in tumor tissue slides from our hospital. Our finding clarified the relationship between risk score and ICB related signatures, which indicated the predictive significance of the model. The potential mechanism of risk score was also explored, which was related to immune-related signal ways. We believed that this model would improve risk stratification in TNBC and serve as a potential immunotherapy biomarker for patients to provide more exact judgment for individualized clinical management.

## Materials and methods

### Study design

**Fig. 1:**
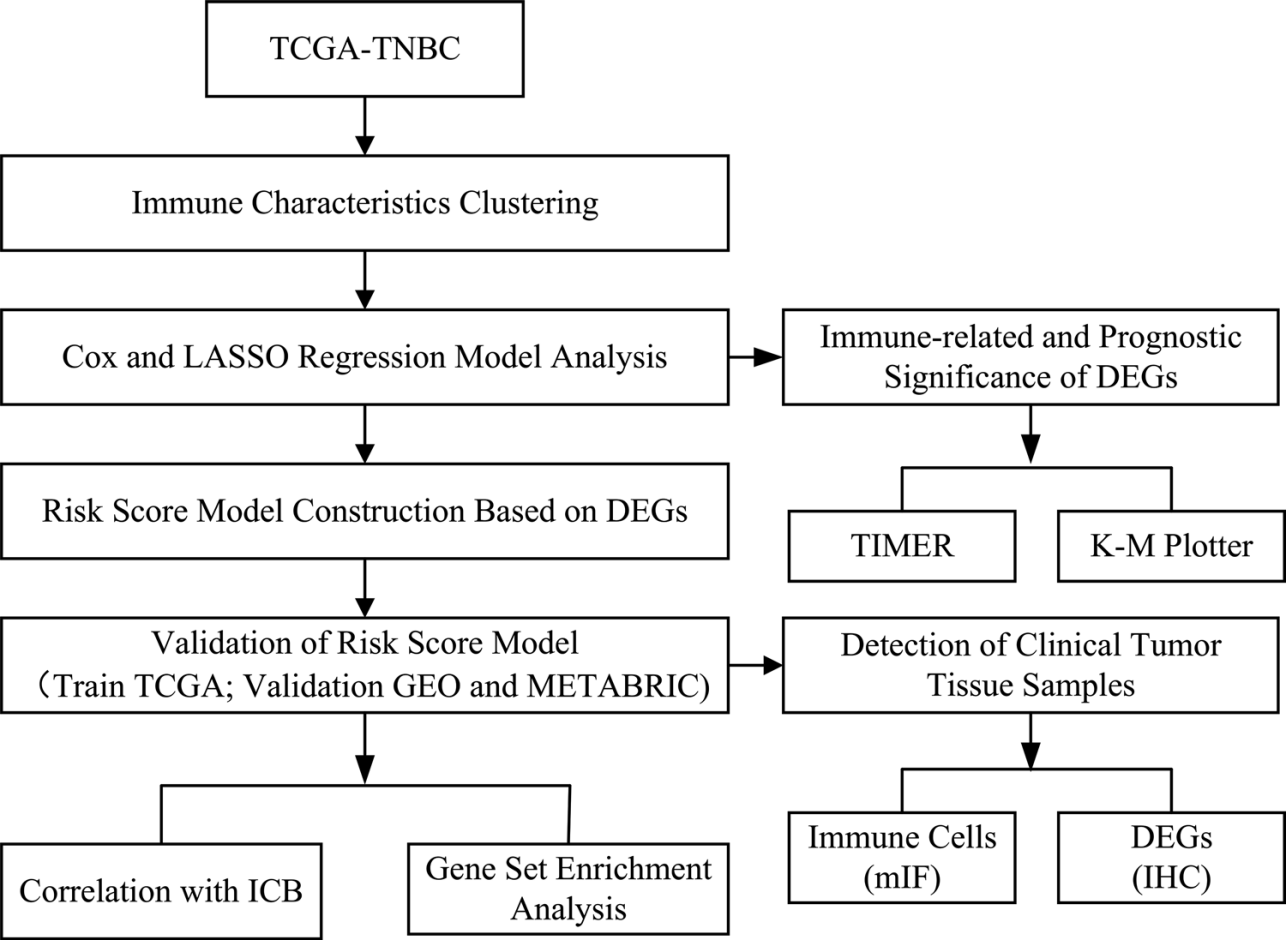
Flow chart of study design

The flow chart of the research is shown in Fig. 1. We analyzed the mRNA profiles of TNBC samples from TCGA databases and clustered them into two different subgroups according to the immune characteristics of TME. Then we performed difference analysis of prognosis and immune between the two groups. By Cox and LASSO analysis, the DEGs were distinguished. Then we evaluated the immune-related and prognostic significance of DEGs from TIMER and K-M plotter website. At the same time, we constructed a risk score model and verified it in GEO and METABRIC database. We also validated the relationship between the model and infiltrating immune cells of TNBC clinical tissue samples using mIF and IHC staining. We acclaimed the predictive value of the model for immunotherapy and prognosis in TNBC. The correlations between the risk score and ICB related signatures were further explored in the TCGA datasets. GSEA was conducted for biological functional exploration of risk score model.

### Database collection

TNBC datasets were downloaded from TCGA database (https://gdc.cancer.gov/), including clinical data and mRNA-seq profiles measured by Illumina HiSeq2000 RNA Sequencing platform. The GSE58812 datasets and the METABRIC datasets were used for validation. The clinical characterizes and mRNA-seq data were downloaded from the GEO portal (https://www.ncbi.nlm.nih.gov/geo/) and cBioPortal (https://www.cbioportal.org/datasets), respectively. In the end, 112 patients with TNBC were enrolled in this research from TCGA database, as well as 107 patients were obtained from GSE58812 datasets and 209 patients were from METABRIC datasets.

### Landscape of immune infiltration in tumor microenvironment

GSVA package was conducted to realize ssGSEA [22] and calculate the enrichment scores of 58,524 genes representing 29 immune associated gene sets of TCGA cohorts. The TNBC samples were stratified into two subgroups, which were high and low immunogenicity groups, based on the immune characteristic clustering using R package, sparcl [23]. We defined them as Immunity-H and Immunity-L group, respectively. Estimation of Stromal and Immune Cells in Malignant Tumors using the Expression Data (ESTIMATE) algorithm was used to calculate the tumor purity, ESTIMATE score, immune score and stromal score of TNBC samples by using R package, estimate. Levels of infiltrating immune cells and stromal cells were predicted, which formed the basis for inferring tumor purity [24]. The CIBERSORT algorithm was used to identify the relative fractions of 22 tumor infiltrating immune cells in each tumor samples, using R package, CIBERSORT. The bar charts of each sample were drawn using R package, barplot [25]. We used multivariate Cox regression analysis to explore the factors associated with TNBC with immunity subgroup and migration status as covariates. Survival comparisons between Immunity-H and Immunity-L group were plotted with R-packages, survival and survminer.

### DEGs between subgroups and construction of risk score model

Based on the previous clustering subgroups, DEGs were determined by using R package, limma. The threshold of DEGs were |log2 fold change (log2FC)|> 2 and false discovery rate (FDR) < 0.05. All results were visualized by heatmap with the R-package, pheatmap. Volcano plot was draw by R package, ggplot. To quantify the effects of immune and prognostic status and minimize overfitting, LASSO regression model using glmnet package in R software was adopted. Based on the optimal lambda value which was selected through a tenfold cross-validation in LASSO method (lambda.min), the optimal DEGs signatures and their LASSO coefficients were obtained [26]. And the risk score was calculated according to the expression level of each gene and its corresponding regression coefficient. The following formula $\left( {risk\;score = \sum\nolimits_{i = 1}^{n} {(Genei \times Coefi)} } \right)$ was performed [27]. Using cox regression model with R package, survival to confirm the prognostic significance of the risk score model, compared with clinical information of TNBC patients.

### The immune-related and prognostic significance of DEGs

TIMER (https://cistrome.shinyapps.io/timer/) was used to confirm the correlation between DEGs and six tumor infiltrating immune cells. Kaplan–Meier Plotter (http://kmplot.com/analysis/) was adopted to analyze the prognosis of DEGs based on Affymetrix microarrays. Hazard ratios (HR) with 95% confidence intervals and log rank *P* value were determined.

### Validation of risk score model

TCGA datasets were set as training group, while GSE58812 and METABRIC datasets were set as validation group to prove the efficiency of risk score model using R package, ggrisk. According to the median risk score, we divided patients into two groups. Then, survival comparisons between high and low risk score groups were visualized by Kaplan–Meier survival curves and examined by log rank test using the R-packages, survival and survminer. ROC curves were developed to estimate the sensitivity and predictive ability of risk score model by R package, timeROC. After the approval of the ethics committee and with the informed consent of the patients, 32 Formalin-fixed paraffin-embedded (FFPE) tissue sections diagnosed as TNBC were collected from the First Affiliated Hospital of Xi'an Jiaotong University during 2015–2020 to analyse the correlation between risk score and immune cells in TME.

### Detection of the fractions of immune cells and expression of DEGs

Various immune cells were tested by multiplex immunofluorescence (mIF) in PerkinElmer Mantra Quantitative Pathology Workstation/Quantitative Pathology Analysis Platform. The detected marker and its corresponding cell type is presented in Table 1. Immunohistochemistry (IHC) was performed on FFPE sections using CXCL13 antibody (rabbit, 1: 200, proteintech), CCL5 antibody (rabbit, 1: 200, Affinity) and GZMB antibody (rabbit, 1: 200, proteintech) according to the manufacturer’s instructions to detect DEGs. The staining was confirmed by two independent investigators. The following method was used to quantify the expression of genes. Firstly, three fields of view were randomly selected and 20 cells were counted in each field of view. The staining results were judged according to the staining intensity and the percentage of positive cells. Staining intensity (SI): negative (no staining) 0 score, weak positive (light yellow) 1 score, medium positive (yellow) 2 score, strong positive (brown) 3 score; and the positive cell percentage (PP): no positive cells 0 score, 1–50% 1 score, 51–100% 2 score. Secondly, multiplying the SI and PP is the final staining score of the field, and the sum of the three fields was taken as the average value.

**Table 1:** Detected marker and its corresponding cell type

Detected	PANCK +	CD8 +	CD56 +	CD68 +	
Marker				HLA-DR +	HLA-DR-
Cell type	Tumor cells	CD8 + T cells	Natural Killer (NK) cells	Macrophages M1	Macrophages M2

### Correlation between immune checkpoint genes and IPS

Six key genes of immune checkpoint blockade therapy were enrolled to analyze the correlation with risk score in TNBC, which were CD274 (PD-L1, programmed death ligand 1), programmed death 1 (PD-1, also known as PDCD1), cytotoxic T-lymphocyte antigen 4 (CTLA-4), T-cell immunoglobulin domain and mucin domain-containing molecule-3 (TIM-3, also known as HAVCR2), indoleamine 2,3-dioxygenase 1 (IDO1) and lymphocyte activating gene 3 (LAG3). The Immunophenoscore (IPS) were calculated on a 0–10 scale based on four main parts (effector cells, immunosuppressive cells, MHC (major histocompatibility complex) molecules, and immunomodulators) related gene expression z-scores using machine learning to determine the immunogenicity. The higher IPS was associated with increased immunogenicity and it has been verified that IPS could predict the response of patients to immunotherapy. The IPS of breast cancer patients were downloaded from The Cancer Immunome Atlas (TCIA) (https://tcia.at/home) [28].

### Gene set enrichment analysis

The GSEA (version 4.1.0) software (https://www.gsea-msigdb.org/gsea/index.jsp) was performed to explore the potential molecular mechanisms between the RiskScore_H and RiskScore_L groups in TNBC. A NOM *P* value < 0.05 and FDR *q* value < 0.25 were considered statistically significant.

#### Statistical analysis

Statistical analyses were performed with R software (version 4.1.2), IBM SPSS Statistic 25.0 and GraphPad Prism 8.0. Differences between two groups were compared using two independent sample *t* tests. Pearson analysis was performed to evaluate the correlation between risk score with ICB related genes. The correlation of risk score with OS was performed by Kaplan–Meier analysis and log rank test.

## Results

### Landscape of immune cell infiltration of TME in TNBC

**Fig. 2:**
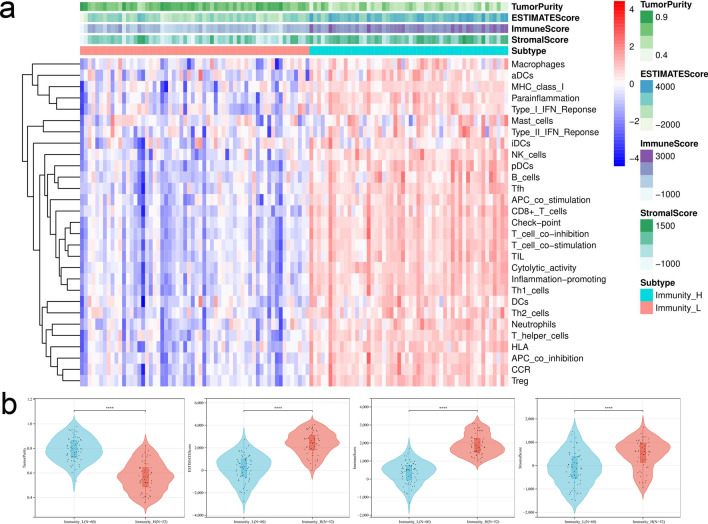
The Landscape of TIME in TNBC. **a** Unsupervised clustering of tumor-infiltrating immune cells with distinction of enrichment of immune scores (ESTIMATEScore, ImmuneScore, StromalScore) and tumor purity in TNBC. **b** Comparison of immune scores and tumor purity between two subgroups. The asterisks represented the statistical *P* value (*P < 0.05; **P < 0.01; ***P < 0.001)

Using ssGSEA algorithm, we calculated 29 immune-related gene sets of TNBC datasets from TCGA database and described the TIME status of TNBC patients. By unsupervised clustering, the samples were divided into two different immune-related subgroups, including Immunity_H group (52 samples) with a high expression level of immune-related gene sets and Immunity_L group (60 samples) with a low expression level of immune-related gene sets. We also calculated the immune scores (ESTIMATEScore, ImmuneScore, StromalScore) and tumor purity between two subgroups using the ESTIMATE algorithm. The involvement of the subgroups with the immune scores and tumor purity was explored and depicted in the comprehensive heatmap (Fig. 2a). It suggested that the immune scores of Immunity_H group were generally higher than those of Immunity_L group, and the tumor purity was lower than that of Immunity_L group (*P* < 0.05; Fig. 2b).

### The immune status and prognostic difference between two subgroups

**Fig. 3:**
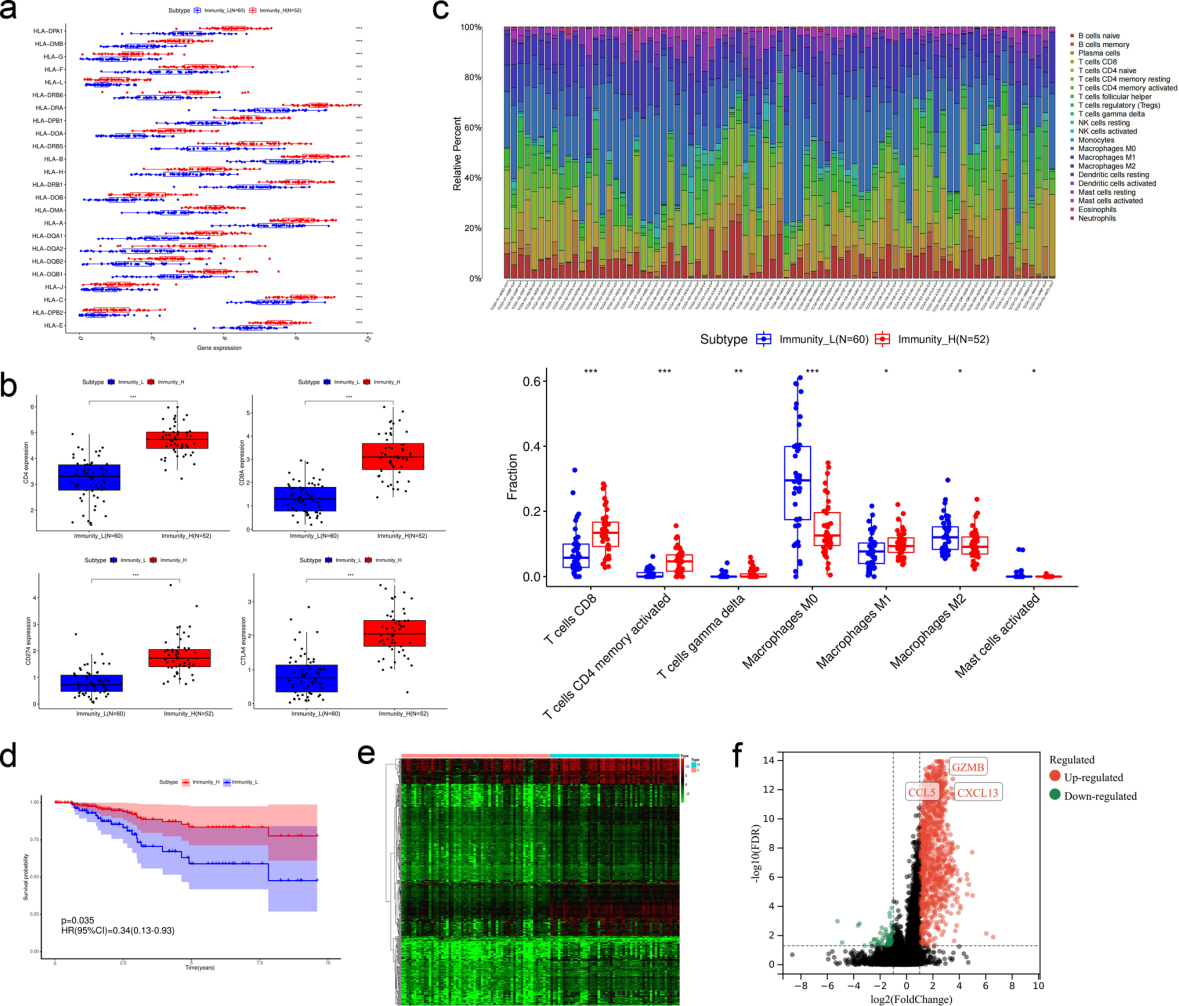
The construction of risk score model. **a** Differences in the expression levels of MHC gene families between two subgroups. **b** CD4, CD8, CD274 (PD-L1) and CTLA4 expression levels between two subgroups. **c** Relative 22 immune cells infiltration in TNBC and the significant differences in the proportion of immune cells between two subgroups. **d** Kaplan–Meier survival curve between two subgroups. **e**, **f** Volcano plot and heat map between two subgroups. The asterisks represented the statistical *P* value (*P < 0.05; **P < 0.01; ***P < 0.001)

In order to further clarify and verify the accuracy of the immune differences between Immunity_H and Immunity_L group, firstly, we compared some immune-related gene expression levels between Immunity_H and Immunity_L group. The expression levels of MHC gene family, CD4, CD8, CD274 (PD-L1) and CTLA4 in Immunity_H group were higher than Immunity_L group (*P* < 0.05; Fig. 3a, b). Meanwhile, the relative subpopulations of infiltrating immune cells were estimated with CIBERSORT approach and the distribution of the proportion of immune cells in TNBC were characterized by bar plot. By comparison, the proportion of immune cells differed between Immunity_H and Immunity_L group. Patients from Immunity_L group exhibited a remarkably higher infiltration of Macrophages M0, Macrophages M2 and activated Mast cells. Immunity_H group was marked by a significant increase in the subpopulation of the antitumor lymphocyte cell subsets, such as CD8 T cells, CD4 memory activated T cells, gamma delta T cells and Macrophages M1 (*P* < 0.05; Fig. 3c). Based on above results, it could be regarded that Immunity_H group was characterized an elevated inflammation response than that of Immunity_ L group. Interestingly, Immunity_H group was characterized by a higher immune score, which indicated an immunologically “hot” phenotype. Previous reports indicated that the cell composition of TME had an important impact on the prognosis of patients, including the degree of immune cells and stromal cells infiltration. In addition, the median overall survival time of Immunity_H group was longer than Immunity_L group (*P* = 0.035, HR = 0.34[0.13–0.93]); Fig. 3d).

### Screening of DEGs and construction of risk score model

**Fig. 4:**
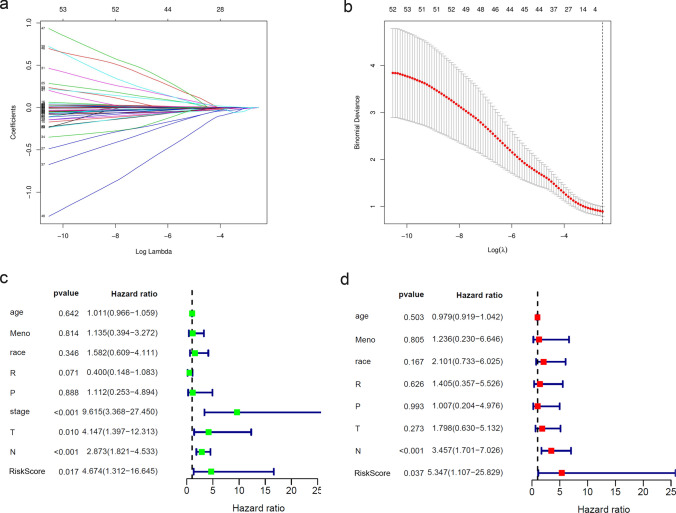
LASSO and Cox risk regression model. **a**, **b** LASSO regression model based on DEGs between two subgroups. **c** Univariate Cox proportional hazard regression analysis between the risk score and OS of TNBC patients. **d** Multivariate Cox proportional hazard regression analysis between the risk score and OS of TNBC patients. (Meno, menopause status; R, radiotherapy; P, physical therapy; T, tumor size; N, lymph node status)

In order to further explore the reasons of the difference in immunogenicity between Immunity_H and Immunity_L group, we analyzed the mRNA-seq data of TNBC patients from TCGA database. And the volcano plot and heat map were drawn to distinguish the DEGs between the two groups (Fig. 3e, f). Combined with LASSO regression (Fig. 4a, b) to reduce the risk of overfitting, 3 optimal predictors with a lambda.min (0.07905125) and log (*λ*) value of − 1.102091258 were chosen to predict immune and prognosis most accurately in TNBC. Three genes with the most significant different expression level were obtained, namely CXCL13, CCL5 and GZMB. And the risk score model was constructed with the following formula: risk score = − 0.0060 × CXCL13 expression − 0.0046 × CCL5 expression − 0.0606 × GZMB expression. Then we calculated the risk score of each sample and combined with its clinical characteristics to analyze the relationship between these various factors and OS by Cox risk regression analysis. Based on the results of the univariate Cox analysis (Fig. 4c), it showed that tumor stage (*P* < 0.001, HR = 9.615[3.368–27.450]), tumor size (*P* = 0.010, HR = 4.147[1.397–12.313]), lymph node status (*P* < 0.001, HR = 2.873[1.821–4.533]) and risk score (*P* = 0.017, HR = 4.674[1.312–16.645]) had a statistically significant impact on patient survival. The results of multivariate Cox analysis (Fig. 4d) revealed that lymph node status (*P* < 0.001, HR = 3.457[1.701–7.026]) and risk score (*P* = 0.037, HR = 5.347[1.107–25.829]) were favorable prognostic factors for survival of TNBC patients. The results suggested that risk score could be an independent prognostic factor in both univariable and multivariable Cox regression analyses.

### The correlation of DEGs with immune infiltration and prognosis

**Fig. 5:**
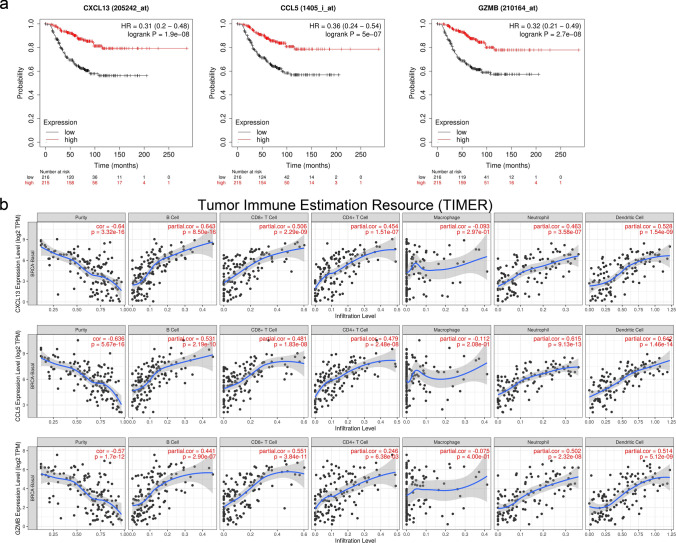
DEGs (CXCL13, CCL5, GZMB) expression levels correlated with immune infiltration and prognosis. **a** Prognostic value of DEGs in TNBC. **b** Expression of DEGs was related to immune infiltration cells in breast cancer

We used the Kaplan–Meier Plotter to evaluate prognostic value of DEGs in TNBC. The results showed that the expression of DEGs was related to OS of TNBC patients, and their expression level was positively correlated with better OS among all TNBC patients (*P* < 0.05; CXCL13, HR = 0.31[0.2–0.48]; CCL5, HR = 0.36[0.24–0.54]; GZMB, HR = 0.31[0.2–0.48]; Fig. 5a). The TIMER database was performed to investigate whether DEGs were related to immune infiltration in breast cancer. The results revealed that the expression level of DEGs was positively correlated with tumor immune infiltration in breast cancer. Except for macrophages, other immune cells had significant statistical differences (*P* < 0.05; Fig. 5b). These results suggested that DEGs may serve as an immune-related tumor marker in breast cancer which could predict the prognosis in TNBC.

### Validation of risk score model based on DEGs in TNBC

**Fig. 6:**
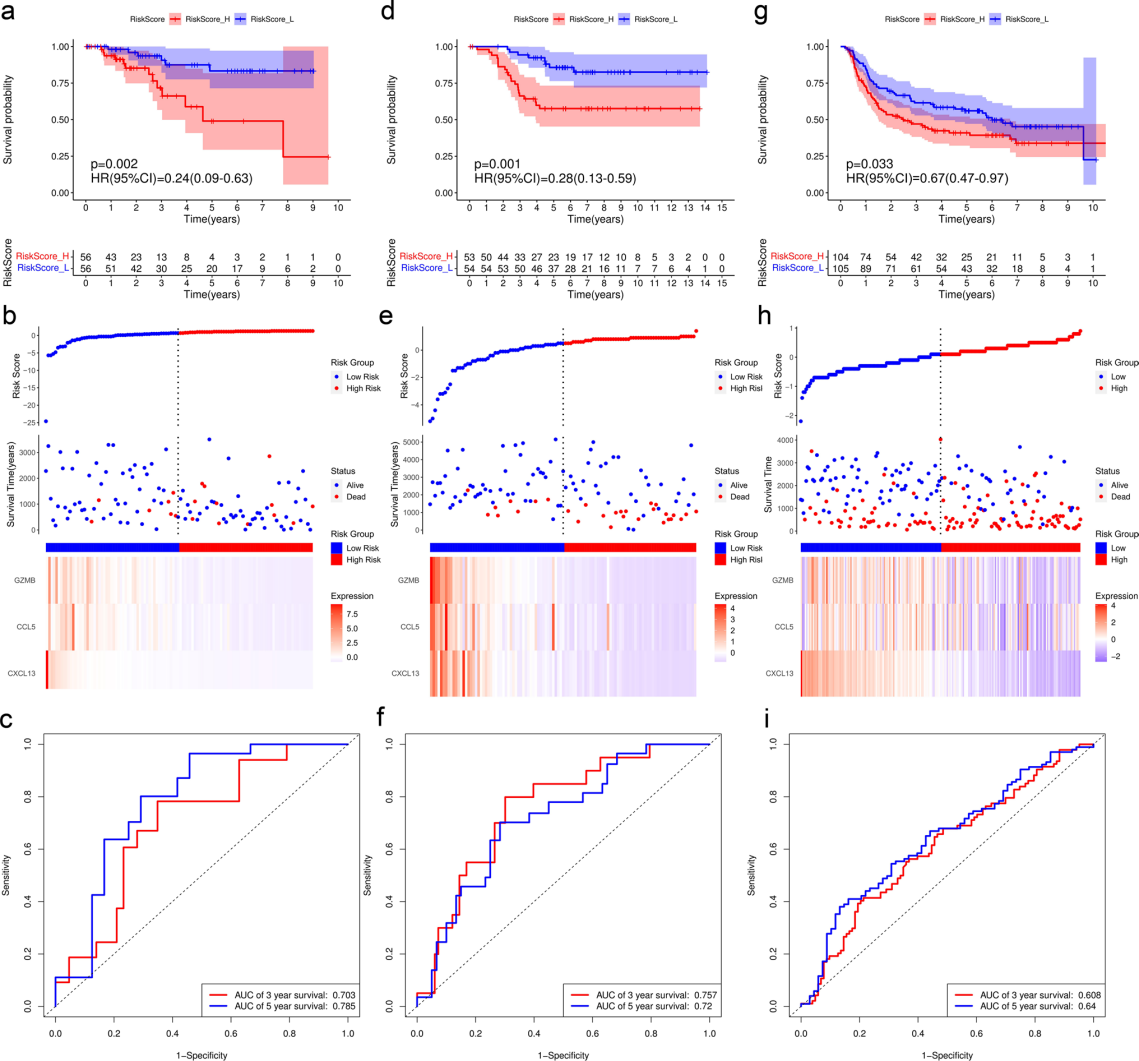
Prognostic value of risk score model based on DEGs. **a–c** TCGA training set. **d**–**f** GEO validation set. **g**–**i** METABRIC validation set. **a**, **d**, **g** Analysis of risk score for TNBC patients with K-M survival curve. **b**, **e**, **h** Distribution plot of gene risk evaluation (top); Survival status of each patient (middle). Heatmap of DEGs expression levels (bottom). **c**, **f**, **i** Time-dependent ROC curve analysis (AUC, area under curve)

In order to further verify the accuracy of the prognostic prediction of risk score model in TNBC, we confirmed the reliability in three TNBC cohorts, including 112 cases from TCGA database as training set (Fig. 6a–c), 107 cases from GEO database (Fig. 6d–f) and 209 cases from METABRIC database as validation sets (Fig. 6g–i). We calculated the risk score of each sample using the risk score model formula (risk score = − 0.0060 × CXCL13 expression − 0.0046 × CCL5 expression − 0.0606 × GZMB expression). According to the median value of risk score, the TNBC patients were divided into RiskScore_L group with low risk score and RiskScore_H group with high risk score. Kaplan–Meier survival curve was performed. The results showed that the prognosis of RiskScore_L group was significantly better than that of RiskScore_H group in all three databases (*P* < 0.05; TCGA, HR = 0.24[0.09–0.63]; GEO, HR = 0.28[0.13–0.59]; METABRIC, HR = 0.67[0.47–0.97]; Fig. 6a, d, g). The ranking was based on the risk score values of the DEGs from low to high, the risk score distribution (Fig. 6b), patient survival status (Fig. 6e) and risk gene expression (Fig. 6h) in sets were shown, respectively. The time-dependent ROC curve analysis of the risk score in three databases revealed the robustness of the prognostic capability of the risk score for OS (TCGA, 3 year survival, AUC = 0.703; 5 year survival, AUC = 0.785; GEO, 3 year survival, AUC = 0.757; 5 year survival, AUC = 0.720; METABRIC, 3 year survival, AUC = 0.608; 5 year survival, AUC = 0.64; Fig. 6c, f,i). The above results indicated that the risk score model based on three immune-related DEGs was validated to be a strong predictive signature on the survival of TNBC patients.

**Fig. 7:**
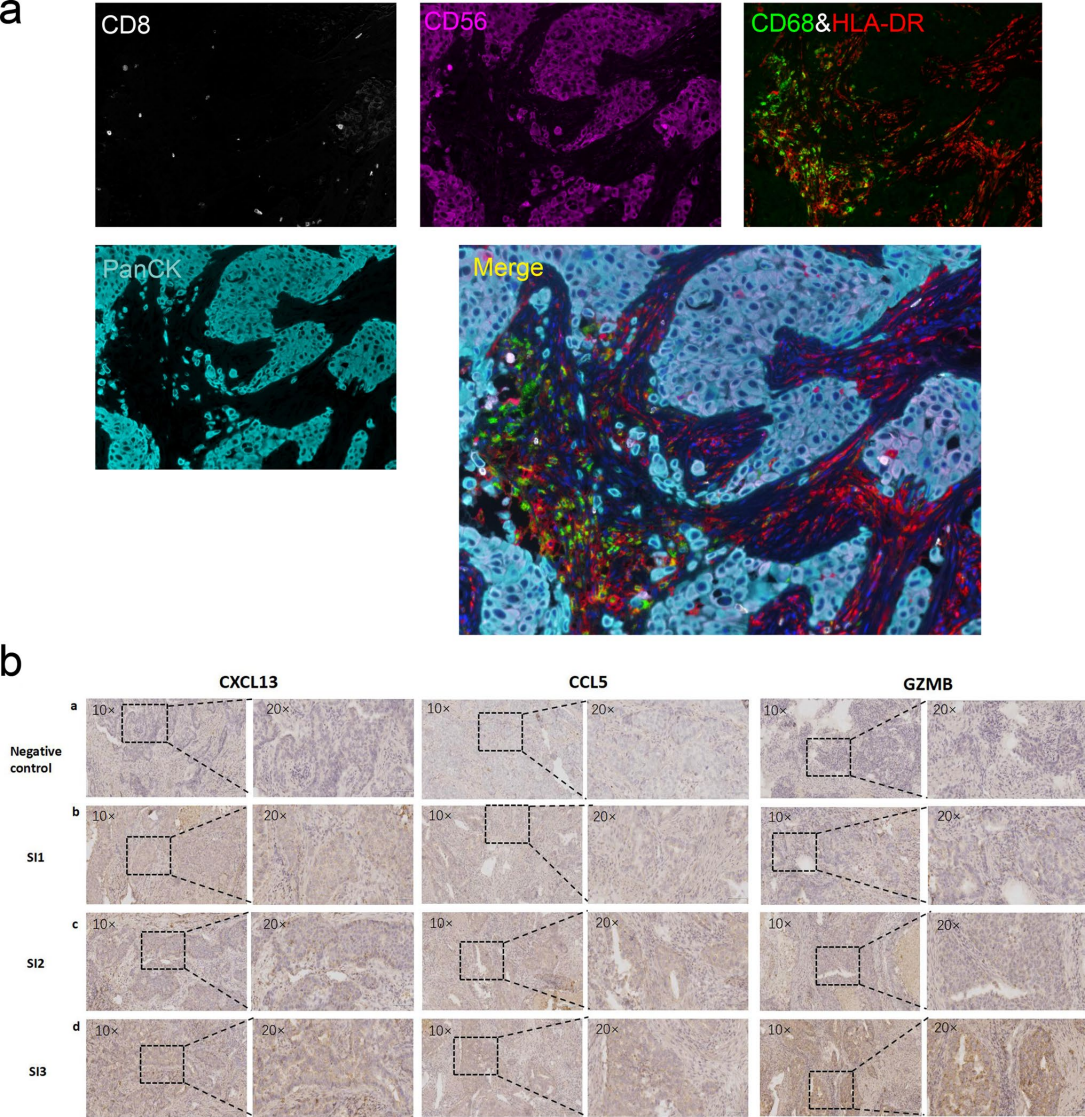
The detection of clinical tissue samples. **a** Immune cells in tumor immune microenvironment (100 ×). Merge (composite) represents the final integrated map after detecting all markers in the tumor microenvironment. Different colors in the figure represent different molecular markers, PanCK (cyan) is the molecular marker of tumor cells, and CD8 (white) is the CD8^+^ T cells. The molecular marker, HLA-DR (red) and CD68 (green) are combined as the molecular marker for macrophages, and CD56 (purple) is the molecular marker for NK cells. **b** DEGs expression levels (10 × & 20 ×). **a** Negative control. **b** SI1 is staining intensity 1 score. **c** SI2 is staining intensity 2 score. **d** SI3 is staining intensity 3 score

We collected the clinical samples from our hospital to identify our study. We detected the immune cells in tumor microenvironment of 32 TNBC pathological tissue specimens using mIF, which included marker PanCK, CD8, HLA-DR, CD68, CD56 (Fig. 7a). The expression levels of three DEGs (CXCL13, CCL5 and GZMB) were performed by IHC staining on TNBC tissue samples (Fig. 7b). The risk score and immune cells fractions of 32 TNBC tumor tissue specimens were calculated (Supplementary Table 1). And the samples were divided into two groups based on the median of risk score. We analyzed the correlation between risk score and the proportions of immune cells in TME. The results displayed that the fractions of immune cells were different in two groups, especially about NK cells and macrophages. Low risk score group was associated with higher proportions of NK cells and macrophages M1/M2 ratio (Supplementary Fig. 1).

### Predictive significance for immunotherapy and Immune-related pathways

**Fig. 8:**
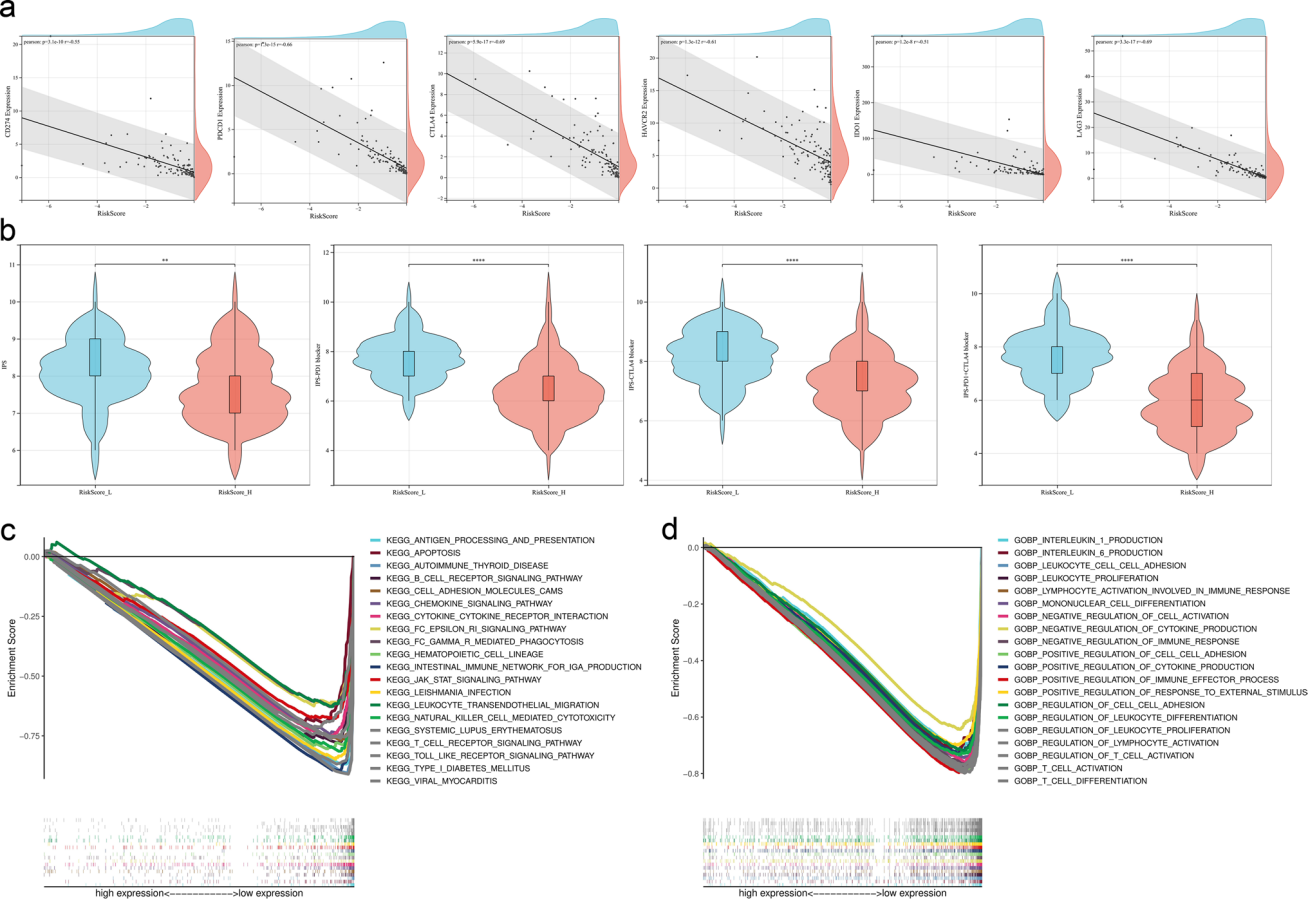
The immunity of risk score. **a** The correlation between risk score with ICB related genes (CD274, PDCD1, CTLA4, HAVCR2, IDO1 and LAG3). The test for association between paired samples used Pearson's correlation coefficient. **b** The IPS between RiskScore_L and RiskScore_H group. The asterisks represented the statistical *P* value (*P < 0.05; **P < 0.01; ***P < 0.001). **c** Top twenty significant pathways associated with low risk score based on KEGG analysis (NOM *P* value < 0.05 and FDR *q* value < 0.25). **d** Top twenty significant pathways associated with low risk score based on GO biological processes analysis (NOM *P* value < 0.05 and FDR *q* value < 0.25)

We checked the correlation between the risk score and six key ICB related genes, including CD274 (PD-L1), PDCD1, CTLA4, HAVCR2, IDO1 and LAG3. We observed that risk score had a remarkably negative relationship to the expression of these ICB related genes (*P* < 0.05; Fig. 8a), which showed that risk score might act as a significant role in the prediction of responsiveness to ICB treatment in TNBC. Furthermore, we confirmed the IPS to predict efficacy of ICB with PD-1 and/or CTLA-4 inhibitors, including the IPS, IPS-PD1 blocker, IPS-CTLA4 blocker and IPS- PD1 + CTLA4 blocker. In our study, the predictive value of risk score for ICB treatment were checked. RiskScore_H group was marked by a significantly lower IPS than RiskScore_L group (*P* < 0.05; Fig. 8b) which suggested that RiskScore_L group might be more suitable for immunotherapy. These results suggested the risk score model might be a potential predictive signature for response to immunotherapy in TNBC. At the same time, we performed GSEA to explore the biological processes related to risk score in TNBC. The top twenty significant pathways with comprehensive details were recorded in Supplementary Table 2. KEGG significant pathways associated with low risk score were mainly involved in immune-related pathways, including antigen processing and presentation, B cell receptor signaling pathway, cytokine-cytokine receptor interaction, chemokine signaling pathway, intestinal immune network for IgA production, leukocyte transendothelial migration, natural killer cell mediated cytotoxicity, T cell receptor signaling pathway (Fig. 8c). GO biological processes significant pathways associated with low risk score were also mainly enriched in immune-related signal pathways, such as IL-1 signaling pathway, IL-6 signaling pathway, leukocyte proliferation, lymphocyte activation involved in immune response, mononuclear cell differentiation, regulation of immune response, T cell activation, T cell differentiation and so on (Fig. 8d). Through GSEA results of risk score, the immune-related pathways were observed obvious enrichment in low risk score group which suggested that risk score was closely linked to immune cell infiltration, inflammatory reactions and TME modification of TNBC.

## Discussion

Immunotherapy has started being considered as one of the emerging and effective treatments in breast cancer, especially for the patients with TNBC [29]. TNBC has high expression levels of TILs, PD-L1, tumor mutational burden, microsatellite instability, mismatch repair genes and other characteristics. These indicators cause the immunogenicity of TNBC which are different from other types of breast cancer. And these might be the reasons why TNBC patients are more sensitive and responsive to immunotherapy. We might can use these biological indicators as efficient predictors of beneficial population in breast cancer immunotherapy [5, 30]. The interaction between tumor cells and immune cells is related to cancer development and progression [31]. In recent years, TILs as an important part of TME have been confirmed as an prominent biomarkers in the prediction of immunotherapy [29]. Therefore, it is necessary to explore more signatures from TIME in TNBC. Recently, the studies of Jérôme GALON group discussed the meaning of the individual immune parameters and combination of multiple immune factors, such as immune cell populations, immune genes, cytokines and so on. They introduced a novel immune-based assay named the ‘Immunoscore’ based on the main immune parameter in cancer with classification, prognostic and predictive value [32].

In this study, we depicted the immune infiltration status of TME with heatmap based on 29 immune-related gene sets of TNBC datasets from TCGA database which were evaluated by ssGSEA algorithm. It is suggested that TNBC had internal immunological heterogeneity which has already been reported [33]. Based on the immune characteristic clustering, all TNBC samples were clustered into two subgroups which were Immunity_L and Immunity_H group. Through ESTIMATE algorithm, we calculated tumor purity, stromal and immune scores of each sample according to immune or stromal components in TME. The patients with TNBC in Immunity_H group were characterized with the abundance of immune scores which presented higher immune infiltration levels and associated with stronger immune activity [34]. Immune cell compositions were analyzed by CIBERSORT algorithm [25]. The infiltration of Macrophages M0, Macrophages M2 and activated Mast cells were enriched in Immunity_L group. On contrary, Immunity_H group obtained more infiltration of CD8 T cells, CD4 memory activated T cells, gamma delta T cells and Macrophages M1. Above all, it indicated that Immunity_H group is an immunologically “hot” phenotype with an elevated inflammation response. We also compared the expression levels of some confirmed immune-related genes (MHC gene family, CD4, CD8, CD274 and CTLA4) [35] between two subgroups. The results showed the significant up-regulated expression of immune-related genes in Immunity_H group than Immunity_L group. As reported before, TME was related to tumorigenesis, which affected the outcome of patients with TNBC [36]. We explored the prognostic value between Immunity_H and Immunity_L group, and we found patients in Immunity_H group were accompanied with a prolonged median OS. Recent studies also indicated that “immune-inflamed” cluster in TNBC was abundant with adaptive and innate immune cells infiltration and high expression of immune checkpoint molecules. And it had significantly better progression free survival (RFS) and OS than other clusters [11] which was similar to our research. In order to discover the cause of the difference between two subgroups, we compared the mRNA expression level of TNBC and the DEGs were distinguished using limma R package. After the application of Cox and LASSO regression analysis, three optimal DEGs were determined which were CXCL13, CCL5 and GZMB.

CXCL13 was a chemokine ligand playing an important role in recruiting lymphocytes (such as naive and effector CD4 + T cells, memory CD4 + T cells, CD8 + T cells, B cells and natural killer cells, etc.) to the site of inflammation [37]. In breast cancer tissues, CXCL13 gene was overexpressed at mRNA level and protein level. And CXCL13 immunohistochemistry marker was related to the degree of tumor immune infiltration [38]. Studies have proved that the expression of CXCL13 gene could predict the increase of TILs and the improvement of disease free survival and OS in early TNBC [39, 40]. CCL5 was a chemokine involved in immune regulation and inflammation [41], and its mRNA expression was significantly related to immune activation and pathologic complete response increase of neoadjuvant chemotherapy in breast cancer (*P* < 0.001, OR = 1.41[1.23–1.62]) [42]. In a retrospective cohort of 72 TNBC patients and a publicly available dataset analysis, there was a direct correlation between TILs and CCL5 expression in TNBC. In addition, CCL5 gene expression was significantly correlated with better RFS (*P* = 0.0012; HR = 0.39[0.22–0.71]) [43]. The GZMB gene encoded a member of protein granzyme subfamily and was a part of S1 family of serine protease peptidases. It was secreted by natural killer cells and cytotoxic T lymphocytes which were related to the apoptosis of target cells, chronic inflammation and wound healing. B cells could kill tumor cells through GZMB [20, 44]. GZMB + TILs were markers of immune escape from primary tumors and were associated with metastatic tumors [45]. In summary, it could be seen that the three genes CXCL13, CCL5 and GZMB were all immune-related prognostic genes in breast cancer. Most of the conclusions tended to be pro-inflammatory. The higher the gene expression level, the better the patient’s prognosis and response to treatment [46]. Our study further proved the expression level of three DEGs were higher in breast cancer tissue than normal breast tissue. Meanwhile, we also confirmed the favorable effect of CXCL13, CCL5 and GZMB on prognosis and the positive correlation with immune activity in TNBC.

Based on above results, a risk score model was constructed. The formula was as follows: risk score = − 0.0060 × CXCL13 expression − 0.0046 × CCL5 expression − 0.0606 × GZMB expression. We confirmed the prognostic significance of the risk score model in univariable and multivariable Cox regression model. It suggested that the risk score could be regard as an independent favorable prognostic factor in TNBC. The predictive capability of the risk score model was also successfully validated in TCGA, GEO and METABRIC databases. The risk score distribution, patient survival status, risk gene expression of three DEGs and ROC curve analysis using risk score were also performed showed a better prognostic value. In all cohorts, patients in low risk score group showed prolonged survival time than high risk score group which verified the model could provide a valuable prognostic signature for patients with TNBC. We suggested the risk score was related to the fractions of different immune cells in TME. Low risk score group had higher proportions of NK cells and macrophages M1/M2 ratio which might be related to a better prognosis. Because of the small clinical enrolled sample size, we did not get the statistical difference. Moreover, other confounders related to our research may have influence on this formula, and samples were not large enough to make results more precise. More attention should be paid when our results were applied for clinical research. In subsequent research, we planned to enroll external patients with TNBC for further exploration at a larger cohort.

Our risk score model derived from TIME and it was related to the survival outcomes in TNBC, so we further explored whether risk score correlated with immunotherapy of TNBC. Refer to existing studies, the expression level of ICB related genes might be related to the efficiency of ICB treatment [47]. Therefore, we analyzed the relationship between risk score and several important ICB related genes, such as CD274, PDCD1, CTLA4, HAVCR2, IDO1 and LAG3. The result showed that risk score was negatively correlated with the expression of ICB related genes. It suggested that the risk score may could predict the response to ICB treatment. Low risk group patients were more likely to respond to immunotherapy. At the same time, we downloaded the IPS which could predict the response of the cancer patients to immunotherapy with anti-PD-1, anti-PD-L1 and/or anti-CTLA-4 treatment from TCIA [48]. We found that IPS of high risk score group was significantly lower than that of low risk score group. In addition, GESA enrichment analysis exhibited that a low risk score showed a mainly significant enrichment in immune-related pathways from KEGG and GO biological processes. Above all, the risk score model might be used as a predictor of immunotherapy in TNBC. We could say that the patients with low risk score showed sensitive responses to ICB treatment.

## Conclusion

In conclusion, TNBC has internal heterogeneity. We distinguished three immune-related prognostic genes from clustering subgroups correlated with TIME. And we built and verified a novel prognostic model based on the three genes. The model could be used in molecular subgrouping and accurately predict the prognosis of TNBC, especially for the evaluation of response to immunotherapy.

### Supplementary Information

Below is the link to the electronic supplementary material.

Supplementary file1 (DOCX 31 KB)

Supplementary file2 (DOCX 23 KB)

Supplementary file3 (DOCX 17 KB)

## Data Availability

The datasets generated and/or analysed during the current study are available in the TCGA repository, [https://gdc.cancer.gov/], GEO portal, [https://www.ncbi.nlm.nih.gov/geo/] and cBioPortal, [https://www.cbioportal.org/datasets]. In addition, The original contributions presented in the study are included in the article/supplementary material. Further inquiries can be directed to the corresponding authors.

## Abbreviations

CTLA-4: Cytotoxic T-lymphocyte antigen 4
DEGs: Differentially expressed genes
ER: Estrogen receptor
ESTIMATE: Estimation of stromal and immune cells in malignant tumors using the expression data
FDR: False discovery rate
FFPE: Formalin-fixed paraffin-embedded
GEO: Gene Expression Omnibus
GSEA: Gene set enrichment analysis
HER-2: Human epidermal grow the factor receptor 2
HR: Hazard ratios
ICB: Immune checkpoint blockades
IDO1: Indoleamine 2,3-dioxygenase 1
IHC: Immunohistochemistry
IPS: Immunophenoscore
LAG3: Lymphocyte activating gene 3
LASSO: Least absolute shrinkage and selector operation
METABRIC: Molecular Taxonomy of Breast Cancer International Consortium
MHC: Major histocompatibility complex
mIF: Multiplex immunofluorescence
OR: Odd ratio
OS: Overall survival
PD-1: Programmed cell death-1
PD-L1: Programmed cell death-ligand 1
PP: Positive cell percentage
RFS: Recurrence free survival
ROC: Receiver operating characteristic
SI: Staining intensity
TCGA: The cancer genome atlas
TCIA: The cancer immunome atlas
TILs: Tumor-infiltrating lymphocytes
TIM-3: T-cell immunoglobulin domain and mucin domain-containing molecule-3
TIME: Tumor immune microenvironment
TME: Tumor microenvironment
TNBC: Triple negative breast cancer

## References

[1] Ferlay J, Forman D, Mathers CD (2012). Breast and cervical cancer in 187 countries between 1980 and 2010. The Lancet.

[2] World Health Organization. (2020). https://www.iarc.fr/faq/latest-global-cancer-data-2020-qa/.

[3] Garrido-Castro AC, Lin NU, Polyak K (2019). Insights into molecular classifications of triple-negative breast cancer: improving patient selection for treatment. Cancer Discov.

[4] Sun X, Luo H, Han C (2021). Identification of a Hypoxia-related molecular classification and hypoxic tumor microenvironment signature for predicting the prognosis of patients with triple-negative breast cancer. Front Oncol.

[5] Bianchini G, Balko JM, Mayer IA (2016). Triple-negative breast cancer: challenges and opportunities of a heterogeneous disease. Nat Rev Clin Oncol.

[6] Lehmann BD, Bauer JA, Chen X (2011). Identification of human triple-negative breast cancer subtypes and preclinical models for selection of targeted therapies. J Clin Invest.

[7] Wu SY, Wang H, Shao ZM (2021). Triple-negative breast cancer: new treatment strategies in the era of precision medicine. Sci China Life Sci.

[8] Burstein MD, Tsimelzon A, Poage GM (2015). Comprehensive genomic analysis identifies novel subtypes and targets of triple-negative breast cancer. Clin Cancer Res.

[9] He Y, Jiang Z, Chen C (2018). Classification of triple-negative breast cancers based on Immunogenomic profiling. J Exp Clin Cancer Res.

[10] Jiang YZ, Ma D, Suo C (2019). Genomic and transcriptomic landscape of triple-negative breast cancers: subtypes and treatment strategies. Cancer Cell.

[11] Xiao Y, Ma D, Zhao S (2019). Multi-Omics profiling reveals distinct microenvironment characterization and suggests immune escape mechanisms of triple-negative breast cancer. Clin Cancer Res.

[12] Jia H, Truica CI, Wang B (2017). Immunotherapy for triple-negative breast cancer: existing challenges and exciting prospects. Drug Resist Updates.

[13] McArthur HL, Page DB (2016). Immunotherapy for the treatment of breast cancer: checkpoint blockade, cancer vaccines, and future directions in combination immunotherapy. Clin Adv Hematol Oncol.

[14] Schmid P, Cortes J, Pusztai L (2020). Pembrolizumab for early triple-negative breast cancer. N Engl J Med.

[15] Schmid P, Adams S, Rugo HS (2018). Atezolizumab and nab-paclitaxel in advanced triple-negative breast cancer. N Engl J Med.

[16] Marra A, Viale G, Curigliano G (2019). Recent advances in triple negative breast cancer: the immunotherapy era. BMC Med.

[17] Qiu P, Guo Q, Yao Q (2021). Characterization of exosome-related gene risk model to evaluate the tumor immune microenvironment and predict prognosis in triple-negative breast cancer. Front Immunol.

[18] Kennedy LB, Salama AKS (2020). A review of cancer immunotherapy toxicity. CA Cancer J Clin.

[19] Hanahan D, Coussens LM (2012). Accessories to the crime: functions of cells recruited to the tumor microenvironment. Cancer Cell.

[20] Byrne A, Savas P, Sant S (2020). Tissue-resident memory T cells in breast cancer control and immunotherapy responses. Nat Rev Clin Oncol.

[21] Wang S, Xiong Y, Zhang Q (2021). Clinical significance and immunogenomic landscape analyses of the immune cell signature based prognostic model for patients with breast cancer. Brief Bioinform.

[22] Hänzelmann S, Castelo R, Guinney J (2013). GSVA: gene set variation analysis for microarray and RNA-seq data. BMC Bioinform.

[23] Witten DM, Tibshirani R (2010). A framework for feature selection in clustering. J Am Stat Assoc.

[24] Yoshihara K, Shahmoradgoli M, Martínez E (2013). Inferring tumour purity and stromal and immune cell admixture from expression data. Nat Commun.

[25] Newman AM, Liu CL, Green MR (2015). Robust enumeration of cell subsets from tissue expression profiles. Nat Methods.

[26] Tibshirani R (1997). The lasso method for variable selection in the Cox model. Stat Med.

[27] Zheng S, Zou Y, Liang JY (2020). Identification and validation of a combined hypoxia and immune index for triple-negative breast cancer. Mol Oncol.

[28] Charoentong P, Finotello F, Angelova M (2017). Pan-cancer Immunogenomic analyses reveal genotype-immunophenotype relationships and predictors of response to checkpoint blockade. Cell Rep.

[29] Chic N, Brasó-Maristany F, Prat A (2022). Biomarkers of immunotherapy response in breast cancer beyond PD-L1. Breast Cancer Res Treat.

[30] Emens LA (2018). Breast cancer immunotherapy: facts and hopes. Clin Cancer Res.

[31] Baxevanis CN, Fortis SP, Perez SA (2021). The balance between breast cancer and the immune system: challenges for prognosis and clinical benefit from immunotherapies. Semin Cancer Biol.

[32] Bruni D, Angell HK, Galon J (2020). The immune contexture and Immunoscore in cancer prognosis and therapeutic efficacy. Nat Rev Cancer.

[33] Metzger-Filho O, Tutt A, de Azambuja E (2012). Dissecting the heterogeneity of triple-negative breast cancer. J Clin Oncol.

[34] Xu M, Li Y, Li W (2020). Immune and stroma related genes in breast cancer: a comprehensive analysis of tumor microenvironment based on the cancer genome atlas (TCGA) database. Front Med (Lausanne).

[35] Li N, Wang J, Zhan X (2021). Identification of immune-related gene signatures in lung adenocarcinoma and lung squamous cell carcinoma. Front Immunol.

[36] Wang X, Su W, Tang D (2021). An immune-related gene prognostic index for triple-negative breast cancer integrates multiple aspects of tumor-immune microenvironment. Cancers (Basel).

[37] Weinstein AM, Storkus WJ (2015). Therapeutic lymphoid organogenesis in the tumor microenvironment. Adv Cancer Res.

[38] Gu-Trantien C, Loi S, Garaud S (2013). CD4^+^ follicular helper T cell infiltration predicts breast cancer survival. J Clin Invest.

[39] Criscitiello C, Bayar MA, Curigliano G (2018). A gene signature to predict high tumor-infiltrating lymphocytes after neoadjuvant chemotherapy and outcome in patients with triple-negative breast cancer. Ann Oncol.

[40] Razis E, Kalogeras KT, Kotsantis I (2020). The Role of CXCL13 and CXCL9 in early breast cancer. Clin Breast Cancer.

[41] Pinto JA, Araujo J, Cardenas NK (2016). A prognostic signature based on three-genes expression in triple-negative breast tumours with residual disease. NPJ Genom Med.

[42] Denkert C, von Minckwitz G, Brase JC (2015). Tumor-infiltrating lymphocytes and response to neoadjuvant chemotherapy with or without carboplatin in human epidermal growth factor receptor 2-positive and triple-negative primary breast cancers. J Clin Oncol.

[43] Araujo JM, Gomez AC, Aguilar A (2018). Effect of CCL5 expression in the recruitment of immune cells in triple negative breast cancer. Sci Rep.

[44] Arabpour M, Rasolmali R, Talei AR (2019). Granzyme B production by activated B cells derived from breast cancer-draining lymph nodes. Mol Immunol.

[45] Shibutani M, Maeda K, Nagahara H (2018). A comparison of the local immune status between the primary and metastatic tumor in colorectal cancer: a retrospective study. BMC Cancer.

[46] Bedognetti D, Hendrickx W, Marincola FM (2015). Prognostic and predictive immune gene signatures in breast cancer. Curr Opin Oncol.

[47] Shi Z, Shen J, Qiu J (2021). CXCL10 potentiates immune checkpoint blockade therapy in homologous recombination-deficient tumors. Theranostics.

[48] Gui CP, Wei JH, Chen YH (2021). A new thinking: extended application of genomic selection to screen multiomics data for development of novel hypoxia-immune biomarkers and target therapy of clear cell renal cell carcinoma. Brief Bioinform.

