# Supplementary material for: A novel model associated with tumor microenvironment on predicting prognosis and immunotherapy in triple negative breast cancer
Source: Clin Exp Med. 2023 May 23;23(7):3867–81. doi: 10.1007/s10238-023-01090-5 (PMC10618350; doi:10.1007/s10238-023-01090-5)
Supplement: Supplementary file 1 — Supplementary file1 (DOCX 31 KB) [file 10238_2023_1090_MOESM1_ESM.docx]

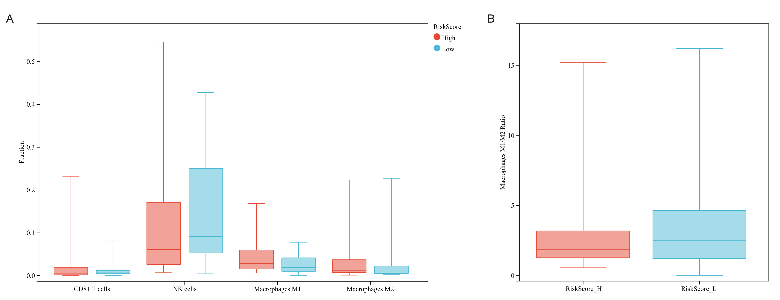


**Supplementary Figure 1** The detection of clinical tissue samples. （A）The correlation between risk score and the proportions of immune cells (CD8+ T cells，NK cells，Macrophages M1 and Macrophages M2) in TME.（B）The macrophages M1/M2 ratio between RiskScore_H and RiskScore_L group.
