# Supplementary material for: A novel model associated with tumor microenvironment on predicting prognosis and immunotherapy in triple negative breast cancer
Source: Clin Exp Med. 2023 May 23;23(7):3867–81. doi: 10.1007/s10238-023-01090-5 (PMC10618350; doi:10.1007/s10238-023-01090-5)
Supplement: Supplementary file 2 — Supplementary file2 (DOCX 23 KB) [file 10238_2023_1090_MOESM2_ESM.docx]

**Supplementary Table 1** The risk score and immune cells fractions of 32 TNBC tumor tissue specimens.

| Detection  Sample | r_PANCK_P | r_CD8_P | r_CD56_P | r_CD68.HLADR_PP | r_CD68.HLADR_PN | r_CD68.HLADR_PP/r_CD68.HLADR_PN | CXCL13 staining | CCL5 staining | GZMB staining | RiskScore | RiskScore Group |
| --- | --- | --- | --- | --- | --- | --- | --- | --- | --- | --- | --- |
|  | Tumor cells | CD8+ T cells | Natural Killer (NK) cells | Macrophages M1 | Macrophages M2 | Macrophages M1/M2 |  |  |  |  |  |
| 1 | 0.3870 | 0.2323 | 0.5460 | 0.1258 | 0.2236 | 0.5627 | 1.3333 | 1.3333 | 2.3333 | -0.1555 | High |
| 2 | 0.3972 | 0.1526 | 0.1920 | 0.1688 | 0.0696 | 2.4270 | 1.6667 | 1.3333 | 1.6667 | -0.1171 | High |
| 3 | 0.4037 | 0.0330 | 0.0824 | 0.0341 | 0.0238 | 1.4317 | 2.0000 | 2.0000 | 2.0000 | -0.1424 | High |
| 4 | 0.3307 | 0.0667 | 0.2489 | 0.0274 | 0.0057 | 4.7778 | 2.0000 | 2.0000 | 3.0000 | -0.2030 | Low |
| 5 | 0.4074 | 0.0099 | 0.2598 | 0.0207 | 0.0049 | 4.2054 | 2.3333 | 1.3333 | 3.0000 | -0.2019 | Low |
| 6 | 0.2615 | 0.0831 | 0.4282 | 0.0781 | 0.0094 | 8.3276 | 5.3333 | 6.0000 | 4.0000 | -0.3020 | Low |
| 7 | 0.5612 | 0.0005 | 0.1732 | 0.0476 | 0.0257 | 1.8507 | 3.3333 | 2.0000 | 3.3333 | -0.2312 | Low |
| 8 | 0.4874 | 0.0189 | 0.4712 | 0.0063 | 0.0079 | 0.7934 | 2.6667 | 1.6667 | 1.6667 | -0.1247 | High |
| 9 | 0.6355 | 0.0065 | 0.1197 | 0.0045 | 0.0213 | 0.2099 | 3.0000 | 2.6667 | 2.6667 | -0.1919 | Low |
| 10 | 0.4826 | 0.0000 | 0.0711 | 0.0082 | 0.0060 | 1.3704 | 1.3333 | 1.3333 | 1.6667 | -0.1151 | High |
| 11 | 0.4204 | 0.0036 | 0.0515 | 0.0444 | 0.0154 | 2.8807 | 1.6667 | 2.6667 | 1.6667 | -0.1233 | High |
| 12 | 0.3587 | 0.0013 | 0.0044 | 0.0173 | 0.0201 | 0.8633 | 1.3333 | 2.6667 | 4.0000 | -0.2627 | Low |
| 13 | 0.4477 | 0.0163 | 0.2551 | 0.0119 | 0.0017 | 6.8519 | 2.3333 | 2.3333 | 1.3333 | -0.1055 | High |
| 14 | 0.4889 | 0.0213 | 0.0069 | 0.0497 | 0.0445 | 1.1180 | 1.6667 | 2.0000 | 2.0000 | -0.1404 | High |
| 15 | 0.1220 | 0.0041 | 0.0170 | 0.0911 | 0.0077 | 11.7615 | 1.3333 | 5.0000 | 1.6667 | -0.1320 | High |
| 16 | 0.4634 | 0.0015 | 0.0291 | 0.0464 | 0.0349 | 1.3306 | 1.6667 | 1.6667 | 1.0000 | -0.0783 | High |
| 17 | 0.4178 | 0.0016 | 0.0140 | 0.0169 | 0.0041 | 4.1239 | 4.0000 | 2.0000 | 1.3333 | -0.1140 | High |
| 18 | 0.3871 | 0.0042 | 0.0686 | 0.0633 | 0.0137 | 4.6094 | 1.6667 | 2.0000 | 2.3333 | -0.1606 | Low |
| 19 | 0.5208 | 0.0009 | 0.1641 | 0.0115 | 0.0123 | 0.9373 | 1.3333 | 1.6667 | 1.3333 | -0.0965 | High |
| 20 | 0.4014 | 0.0112 | 0.0539 | 0.0000 | 0.2276 | 0.0000 | 1.6667 | 4.0000 | 2.3333 | -0.1698 | Low |
| 21 | 0.4123 | 0.0056 | 0.0076 | 0.0120 | 0.0023 | 5.3023 | 2.6667 | 5.3333 | 2.3333 | -0.1819 | Low |
| 22 | 0.4555 | 0.0069 | 0.0943 | 0.0171 | 0.0078 | 2.1804 | 2.6667 | 3.3333 | 2.6667 | -0.1929 | Low |
| 23 | 0.3534 | 0.0139 | 0.4240 | 0.0262 | 0.0070 | 3.7442 | 3.0000 | 2.3333 | 2.6667 | -0.1903 | Low |
| 24 | 0.5380 | 0.0004 | 0.0510 | 0.0043 | 0.0032 | 1.3333 | 4.0000 | 3.0000 | 2.0000 | -0.1590 | Low |
| 25 | 0.5821 | 0.0063 | 0.0313 | 0.0190 | 0.0092 | 2.0598 | 1.6667 | 2.3333 | 1.0000 | -0.0813 | High |
| 26 | 0.2863 | 0.0055 | 0.0139 | 0.0531 | 0.0336 | 1.5803 | 1.6667 | 2.0000 | 3.3333 | -0.2212 | Low |
| 27 | 0.3760 | 0.0138 | 0.0537 | 0.0398 | 0.0025 | 16.2321 | 2.3333 | 1.3333 | 2.6667 | -0.1817 | Low |
| 28 | 0.4371 | 0.0048 | 0.2545 | 0.0137 | 0.0048 | 2.8651 | 4.0000 | 1.6667 | 2.6667 | -0.1933 | Low |
| 29 | 0.3726 | 0.0054 | 0.0114 | 0.0177 | 0.0012 | 15.2381 | 1.0000 | 1.3333 | 1.6667 | -0.1131 | High |
| 30 | 0.2464 | 0.0187 | 0.1644 | 0.1402 | 0.0825 | 1.6995 | 1.6667 | 1.6667 | 1.3333 | -0.0985 | High |
| 31 | 0.4361 | 0.0022 | 0.0415 | 0.0226 | 0.0110 | 2.0604 | 1.0000 | 1.3333 | 1.0000 | -0.0727 | High |
| 32 | 0.4298 | 0.0074 | 0.0886 | 0.0000 | 0.0703 | 0.0000 | 1.3333 | 2.3333 | 2.3333 | -0.1601 | Low |

RiskScore= -0.0060×CXCL13 expression (staining) -0.0046×CCL5 expression (staining)-0.0606×GZMB expression (staining); P, positive; N, negative.
