# Supplementary material for: A novel model associated with tumor microenvironment on predicting prognosis and immunotherapy in triple negative breast cancer
Source: Clin Exp Med. 2023 May 23;23(7):3867–81. doi: 10.1007/s10238-023-01090-5 (PMC10618350; doi:10.1007/s10238-023-01090-5)
Supplement: Supplementary file 3 — Supplementary file3 (DOCX 17 KB) [file 10238_2023_1090_MOESM3_ESM.docx]

**Supplementary Table 2** Top twenty significant pathways associated with low risk score based on GSEA.

| **Supplementary Table 2 Top twenty significant pathways associated with low risk score based on KEGG analysis.** | | | |
| --- | --- | --- | --- |
| **Gene set name** | **NES** | **P-value** | **FDR q-value** |
| KEGG_NATURAL_KILLER_CELL_MEDIATED_CYTOTOXICITY | -2.5021517 | 0 | 0 |
| KEGG_CYTOKINE_CYTOKINE_RECEPTOR_INTERACTION | -2.4672291 | 0 | 0 |
| KEGG_B_CELL_RECEPTOR_SIGNALING_PATHWAY | -2.4125865 | 0 | 0 |
| KEGG_CHEMOKINE_SIGNALING_PATHWAY | -2.3935475 | 0 | 0 |
| KEGG_CELL_ADHESION_MOLECULES_CAMS | -2.3822231 | 0 | 0 |
| KEGG_FC_GAMMA_R_MEDIATED_PHAGOCYTOSIS | -2.2620893 | 0 | 8.66E-05 |
| KEGG_HEMATOPOIETIC_CELL_LINEAGE | -2.2635353 | 0 | 9.32E-05 |
| KEGG_LEISHMANIA_INFECTION | -2.2984428 | 0 | 1.01E-04 |
| KEGG_TOLL_LIKE_RECEPTOR_SIGNALING_PATHWAY | -2.3070197 | 0 | 1.10E-04 |
| KEGG_AUTOIMMUNE_THYROID_DISEASE | -2.3106046 | 0 | 1.21E-04 |
| KEGG_T_CELL_RECEPTOR_SIGNALING_PATHWAY | -2.3140059 | 0 | 1.35E-04 |
| KEGG_JAK_STAT_SIGNALING_PATHWAY | -2.316664 | 0 | 1.51E-04 |
| KEGG_FC_EPSILON_RI_SIGNALING_PATHWAY | -2.2058103 | 0 | 1.57E-04 |
| KEGG_SYSTEMIC_LUPUS_ERYTHEMATOSUS | -2.2324798 | 0 | 1.67E-04 |
| KEGG_ANTIGEN_PROCESSING_AND_PRESENTATION | -2.326152 | 0 | 1.73E-04 |
| KEGG_VIRAL_MYOCARDITIS | -2.2373705 | 0 | 1.78E-04 |
| KEGG_TYPE_I_DIABETES_MELLITUS | -2.1331651 | 0 | 1.88E-04 |
| KEGG_INTESTINAL_IMMUNE_NETWORK_FOR_IGA_PRODUCTION | -2.135329 | 0 | 1.96E-04 |
| KEGG_APOPTOSIS | -2.352452 | 0 | 2.02E-04 |
| KEGG_LEUKOCYTE_TRANSENDOTHELIAL_MIGRATION | -2.140764 | 0 | 2.05E-04 |
| **Supplementary Table 2** Top twenty significant pathways associated with low risk score based on GOBP analysis. | | | |
| **Gene set name** | **NES** | **P-value** | **FDR q-value** |
| GOBP_INTERLEUKIN_1_PRODUCTION | -2.5178366 | 0 | 0 |
| GOBP_INTERLEUKIN_6_PRODUCTION | -2.5082915 | 0 | 0 |
| GOBP_LEUKOCYTE_CELL_CELL_ADHESION | -2.5213969 | 0 | 0 |
| GOBP_LEUKOCYTE_PROLIFERATION | -2.5709507 | 0 | 0 |
| GOBP_LYMPHOCYTE_ACTIVATION_INVOLVED_IN_IMMUNE_RESPONSE | -2.5630367 | 0 | 0 |
| GOBP_MONONUCLEAR_CELL_DIFFERENTIATION | -2.562115 | 0 | 0 |
| GOBP_NEGATIVE_REGULATION_OF_CELL_ACTIVATION | -2.5257285 | 0 | 0 |
| GOBP_NEGATIVE_REGULATION_OF_CYTOKINE_PRODUCTION | -2.5097728 | 0 | 0 |
| GOBP_NEGATIVE_REGULATION_OF_IMMUNE_SYSTEM_PROCESS | -2.5672507 | 0 | 0 |
| GOBP_POSITIVE_REGULATION_OF_CELL_CELL_ADHESION | -2.506086 | 0 | 0 |
| GOBP_POSITIVE_REGULATION_OF_CYTOKINE_PRODUCTION | -2.5648386 | 0 | 0 |
| GOBP_POSITIVE_REGULATION_OF_IMMUNE_EFFECTOR_PROCESS | -2.5041895 | 0 | 0 |
| GOBP_POSITIVE_REGULATION_OF_RESPONSE_TO_EXTERNAL_STIMULUS | -2.5459971 | 0 | 0 |
| GOBP_REGULATION_OF_CELL_CELL_ADHESION | -2.5042024 | 0 | 0 |
| GOBP_REGULATION_OF_LEUKOCYTE_DIFFERENTIATION | -2.5262167 | 0 | 0 |
| GOBP_REGULATION_OF_LEUKOCYTE_PROLIFERATION | -2.507034 | 0 | 0 |
| GOBP_REGULATION_OF_LYMPHOCYTE_ACTIVATION | -2.539504 | 0 | 0 |
| GOBP_REGULATION_OF_T_CELL_ACTIVATION | -2.5245557 | 0 | 0 |
| GOBP_T_CELL_ACTIVATION | -2.5385962 | 0 | 0 |
| GOBP_T_CELL_DIFFERENTIATION | -2.5095124 | 0 | 0 |

NES, normalized enrichment score; FDR, false discovery rate.
